# Direct and indirect evidence of efficacy and safety of rapid exercise tests for exertional desaturation in Covid-19: a rapid systematic review

**DOI:** 10.1186/s13643-021-01620-w

**Published:** 2021-03-16

**Authors:** Asli Kalin, Babak Javid, Matthew Knight, Matt Inada-Kim, Trisha Greenhalgh

**Affiliations:** 1 Department of Primary Health Care Sciences, University of Oxford, Oxford, UK; 2 Division of Experimental Medicine, University of California, Berkeley, CA USA; 3 West Hertfordshire Hospitals NHS Trust, Vicarage Rd, Watford, Hertfordshire, WD18 0HB UK; 4 Hampshire Hospitals NHS Foundation Trust, Basingstoke, UK

**Keywords:** Covid-19, Desaturation, 1-min sit-to-stand test, 6-min walk test, 40-step test, Normoxia, Silent hypoxia, Hidden hypoxia

## Abstract

**Background:**

Even when resting pulse oximetry is normal in the patient with acute Covid-19, hypoxia can manifest on exertion. We summarise the literature on the performance of different rapid tests for exertional desaturation and draw on this evidence base to provide guidance in the context of acute Covid-19.

**Main research questions:**

1. What exercise tests have been used to assess exertional hypoxia at home or in an ambulatory setting in the context of Covid-19 and to what extent have they been validated?
2. What exercise tests have been used to assess exertional hypoxia in other lung conditions, to what extent have they been validated and what is the applicability of these studies to acute Covid-19?

**Method:**

AMED, CINAHL, EMBASE MEDLINE, Cochrane and PubMed using LitCovid, Scholar and Google databases were searched to September 2020. Studies where participants had Covid-19 or another lung disease and underwent any form of exercise test which was compared to a reference standard were eligible. Risk of bias was assessed using QUADAS 2. A protocol for the review was published on the Medrxiv database.

**Results:**

Of 47 relevant papers, 15 were empirical studies, of which 11 described an attempt to validate one or more exercise desaturation tests in lung diseases other than Covid-19. In all but one of these, methodological quality was poor or impossible to fully assess. None had been designed as a formal validation study (most used simple tests of correlation). Only one validation study (comparing a 1-min sit-to-stand test [1MSTST] with reference to the 6-min walk test [6MWT] in 107 patients with interstitial lung disease) contained sufficient raw data for us to calculate the sensitivity (88%), specificity (81%) and positive and negative predictive value (79% and 89% respectively) of the 1MSTST. The other 4 empirical studies included two predictive studies on patients with Covid-19, and two on HIV-positive patients with suspected pneumocystis pneumonia. We found no studies on the 40-step walk test (a less demanding test that is widely used in clinical practice to assess Covid-19 patients). Heterogeneity of study design precluded meta-analysis.

**Discussion:**

Exertional desaturation tests have not yet been validated in patients with (or suspected of having) Covid-19. A stronger evidence base exists for the diagnostic accuracy of the 1MSTST in chronic long-term pulmonary disease; the relative intensity of this test may raise safety concerns in remote consultations or unstable patients. The less strenuous 40-step walk test should be urgently evaluated.

**Supplementary Information:**

The online version contains supplementary material available at 10.1186/s13643-021-01620-w.

## Background

A substantial proportion of patients with acute coronavirus 19 (Covid-19) develop a potentially critical form of the illness requiring intensive care unit admission [1]. The degree of lung involvement in acute Covid-19 is variable, producing a spectrum of illness from mild upper respiratory tract symptoms to acute respiratory distress syndrome [2]. Patients with mild initial symptoms can rapidly deteriorate to severe or critical cases. In hospitalised patients, hypoxaemia and the need for oxygen are independent predictors of severe outcomes [3, 4]. The usual time from symptom onset to the development of severe hypoxemia is between 7 and 12 days [5, 6]. Recent prognostic tools such as the 4C score have emphasised the importance of identifying hypoxia early [3, 7] and there are physiological reasons for managing this hypoxia actively [8, 9].

The poor correlation between both subjective feeling of shortness of breath (dyspnoea) and objective measures of breathlessness and hypoxia in patients with Covid-19 has resulted in UK guidelines recommending that the assessment of the breathless, unwell or high-risk patient should include oximetry [5]. For example, a retrospective cohort study of 64 Covid-19 patients considered eligible for home oximetry monitoring showed that the presence of dyspnoea had a positive predictive value of only 42% for hypoxemia and absence of dyspnoea had a negative predictive value of 86% for excluding it [10].

Indeed, the mismatch between relatively mild subjective respiratory distress and objective evidence of peripheral hypoxia is now recognised as a feature of Covid-19 and has been termed ‘silent’ or ‘happy’ hypoxemia [11, 12]. Similarities with PCP have been drawn by others, and whilst a detailed discussion of pulmonary physiology is outside the scope of this literature review, this feature has been attributed to moderate to severe ventilation-perfusion mismatch [11], intra-pulmonary shunting, loss of lung perfusion regulation, intravascular microthrombi or reduced lung compliance [12, 13].

Hypoxia is common in acute severe Covid-19. Richardson et al. found that 28% of 5700 patients admitted to hospital with acute Covid-19 in New York City were sufficiently hypoxemic to need supplemental oxygen on admission [14]. Yet dyspnoea was reported in only 18.7% of 1099 hospitalised Covid-19 patients, despite low PaO_2_/FiO_2_ ratios and requirement for supplemental oxygen in 41% [15]. In a study of the 19 worst-affected countries worldwide, Goyal et al. reported that rates of mortality were significantly higher in those countries where policies recommended lower oxygen saturations before administering supplemental oxygen than in those that aimed to normalised saturations, though there may have been other explanations for these differences including different degrees of preparedness for a pandemic and different incidence rates [16].

It is widely reported that some patients with acute Covid-19 have normal pulse oximetry at rest but their readings deteriorate on exertion (unpublished data). UK national guidance, for example, recommends the use of exertional desaturation tests to detect early deterioration of patients with COVID-19 in community and emergency settings [17–19]. In consensus exercises, front-line clinicians have identified the late transfer of patients with exertional desaturation (i.e. a fall of 3% or more in pulse oximetry reading on exercise) as a possibly remediable cause of poor outcome [20].

Several publications recommend or suggest the use of exertional tests in the assessment of COVID-19 patients. Mantha et al. discuss the use of a modified 6-min walk test (6MWT), wearing masks and only in those under 70, on day 4 or 5 of clinical illness to risk-stratify patients [6]. This suggestion is echoed by Noormohammadpour and Abolhasani, who propose that deterioration in the 6MWT test can be used to identify those who need referral to hospital [21]. Pandit et al. recommend the 6MWT for those under 60 who are not short of breath at rest and a 3-min walk test (3MWT) for those over 60 who are unable to complete the longer test, but warn that the test is contra-indicated in patients who are hypoxic at rest (SpO_2_ < 94%), short of breath at rest, not able to walk unassisted, have Eisenmenger’s syndrome, severe anaemia, unstable angina or valvular heart disease [2]. They suggest that the 6MWT or 3MWT test can be performed at home or in hospital under the supervision of either a family member or a healthcare professional. These authors define exertional hypoxia as an absolute drop in oximeter reading by 3% or more from baseline. The level of 3% reduction from normal levels is drawn from the British Thoracic Society emergency oxygen guidelines [22] consensus amongst clinicians and a recent empirical study [23].

In sum, consensus guidance and editorials recommend tests for exertional hypoxia in Covid-19, but the evidence base for these tests has not previously been formally reviewed. Indeed, the prognostic utility of exertional desaturation remains unknown. Another unknown is the safety of such tests in patients with suspected Covid-19, especially when used remotely without a clinician physically present. Establishing a validated tool to assess exertional desaturation will help to ensure that future research on this topic can be undertaken in a consistent way.

### Review objective and research questions

The overall objective of this rapid review was to examine the published evidence base for the use of rapid exercise tests and assess their application to the assessment of patients with acute Covid-19. We were particularly interested in tests that could be used outside of the hospital setting, since the reality of acute Covid-19 often involves a remote assessment (with the patient at home at a distance from the clinician) or one in a bespoke ambulatory setting such as a ‘hot hub’ (where exercise tests may be performed outside in car parks, for example, for infection control reasons). We were, however, aware that when we began this study, little or no direct research evidence existed on the use of these tests in patients with Covid-19.

The research questions were:

1. What exercise tests have been used to assess exertional hypoxia at home or in an ambulatory setting in the context of Covid-19 and to what extent have they been validated?
2. What exercise tests have been used to assess exertional hypoxia in other lung conditions, to what extent have they been validated and what is the applicability of these studies to acute Covid-19?

## Methods

We followed Cochrane Rapid Review guidelines [24] and reported our findings using the updated version of the Preferred Reporting Items for a Systematic Review and Meta-analysis of Diagnostic Test Accuracy Studies (PRISMA-DTA) checklist [25]; this is appended as Supplementary table S1. Our team included experienced health librarians as well as systematic reviewers and clinicians (including a general practitioner, general physician, respiratory consultant and emergency medicine consultant with experience of undertaking exercise tests). References were downloaded to Endnote (version 9.0) to maintain and manage citations and facilitate the review process. A protocol for the review was published on the Medrxiv database [26].

### Search strategy and selection criteria

Three independent searches were conducted. The first searched LitCovid, Scholar and Google using the terms ‘step test or field test’ and ‘hypoxia or exertional desaturation’. From these results, promising articles were then used to find additional documents via two methods—forward and backward citation matching, and searching for related articles (also using Microsoft Academic Search).

The second search covered the following databases: AMED, CINAHL, EMBASE MEDLINE and PubMed using the over-arching question ‘Very short exercise desaturation tests for use in the emergency department’. Search terms were:

- (quick OR short) AND oxygen) AND exercise) AND (desaturation OR saturation)) AND test*(‘emergency department*’ AND oxygen) AND exercise) AND desaturation) AND test*)
- (‘emergency department*’ AND oxygen) AND exercise) AND saturation) AND test*)
- (‘emergency department*’ AND oxygen) AND exercise) AND desaturation)
- (‘emergency department*’ AND oxygen) AND exercise) AND saturation)
- (1-min sit-to-stand test) or (‘1 min sit to stand test’) or (‘one minute sit to stand’)

The third search involved searching the Cochrane Database of Systematic Reviews (CDSR), the Cochrane Central Register of Controlled Trials (CENTRAL) and the Cochrane COVID-19 study register.

Search strings for different searches are listed below.

Search of Cochrane Library—Issue 10 of 12, October 2020:

- #1 ((‘step test’ OR ‘walk test’ OR ‘field test’ OR ‘chair rise’ OR ‘sit to stand’ OR ‘exercise test’ OR ‘exercise testing’)):ti,ab,kw OR (exertional NEAR/2 (desaturation OR hypoxia)):ti,ab,kw
- #2 MeSH descriptor: [Coronavirus Infections] this term only
- #3 (coronavirus OR covid*):ti,ab,kw
- #4 #2 or #3
- #5 #1 AND #4

Search of Cochrane COVID-19 study register:

- ‘step test’ OR ‘walk test’ OR ‘field test’ OR ‘chair rise’ OR ‘sit to stand’ OR ‘exercise test’ OR ‘exercise testing’

Our inclusion criteria used a framework based on the PRISMA-DTA standards [25] as follows:

- *Target condition*: Individuals with suspected Covid-19 or another lung disease with or without symptoms.
- *Index test*: Any form of rapid exercise test performed at home or in a healthcare setting.
- *Reference standards*: 6MWT or CPET (which are the most commonly used in exercise testing), or a diagnostic test to diagnose the disease in question such as bronchoalveolar lavage to diagnose pneumocystis pneumonia.
- *Study designs*: clinical practice guidelines and systematic reviews addressing desaturation in exercise tests in lung disease, using the Cochrane definition of a Systematic Review [27]. Primary human studies of all designs (e.g. experimental studies, quasi-experimental studies, diagnostic accuracy studies and observational studies), excluding case series, that involved patients with COVID-19 or a lung disease undergoing rapid exercise testing were included. Editorials and letters around the subject of Covid-19 and rapid exercise testing were included to provide background and surface hypotheses on this new disease.
- *Outcome*. Pulse oximetry or arterial blood gas measurement, and association with any adverse outcome, e.g. hospital admission, need for organ support, death.
- *Time periods*: All periods of time and duration of follow up.

No other limitations were imposed on the search or study selection process. Both peer-reviewed and preprint papers were potentially eligible for inclusion. We planned to seek translation of any relevant papers published in languages other than English, but since none were found, the review was limited to English-language papers. Screening, data abstraction and quality appraisal of full-text papers were completed independently by two reviewers including a topic expert and a review expert. Disagreements were resolved by a consensus-based discussion.

Data elements were extracted from each study onto an Excel spreadsheet, including information on study population and size, study design, inclusion and exclusion criteria, research question, exercise tests used, results and authors’ conclusion. Markers of study quality from each of the articles were identified in the literature search. All extracted data were included in a series of detailed tables and a summary table. There was no requirement to contact authors for missing data.

### Risk of bias appraisal

The risk of bias in individual studies was assessed using the QUADAS 2 tool. Using the signalling questions, AK rated each potential source of bias as high, low or unclear; these ratings were checked independently by TG (see Table 1) [28].

**Table 1:**
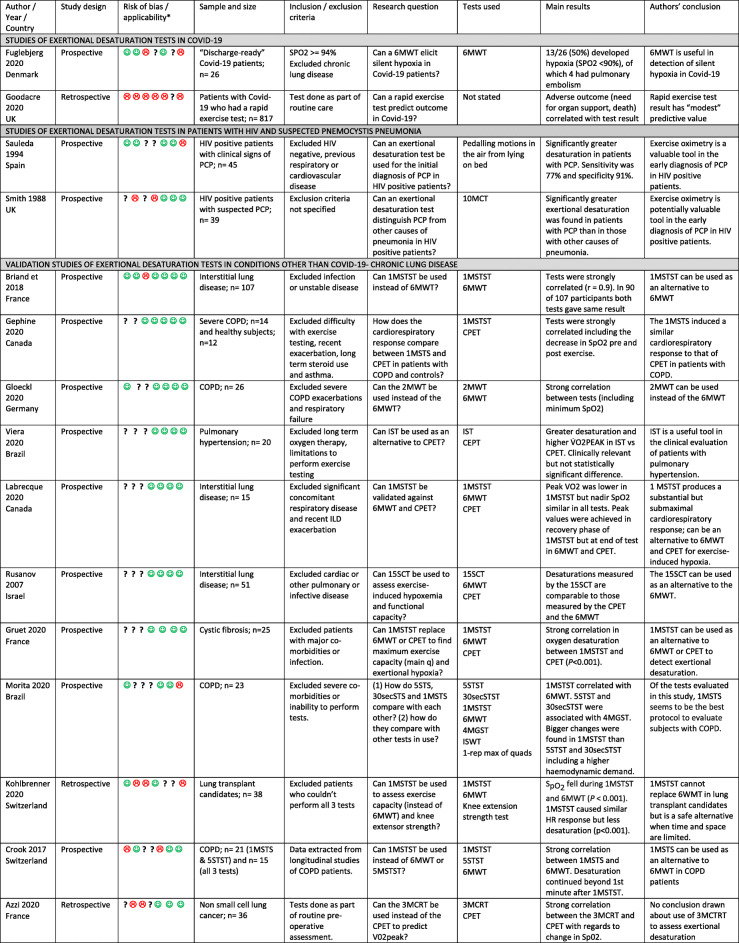
Summary of included empirical studies

*6MWT* 6-minute walk test, *2MWT* 2-minute walk test, *5STST* 5-repetition sit to stand test, *30secSTST* 30 s sit to stand test, *1MSTST* 1-minute STS test, *ISWT* incremental shuttle walking test, *1*-*rep max of quads* 1-repetition maximum of quadriceps muscle, *CPET* cardiopulmonary exercise testing, *15SCT* 15-steps climbing test, *IST* incremental step test, *10MCT* 10-minutes cycling test, *4MGS* 4-minute gait speed test, *3MCTRT* 3-minute chair to rise test, *PCP* pneumocystis pneumonia, *IPF* idiopathic pulmonary fibrosis (IPF)

*Risk of bias column summarises the following assessments in this order: *Risk of bias*: patient selection/index test/reference standard/flow and timing, *Concerns about applicability*: patient selection/index test/reference standard: ; ; ? unclear risk of bias

### Diagnostic accuracy measures

Where relevant, diagnostic accuracy measures are reported as sensitivity, specificity, positive and negative predictive values of the new test (index test) in relation to the gold standard (reference test).

### Synthesis

Given the heterogeneity of the evidence included, formal statistical synthesis was not possible. Data from the included studies were tabulated in summary tables of origins, methods and results, and then summarised narratively.

## Results

### Overview of dataset

**Fig. 1:**
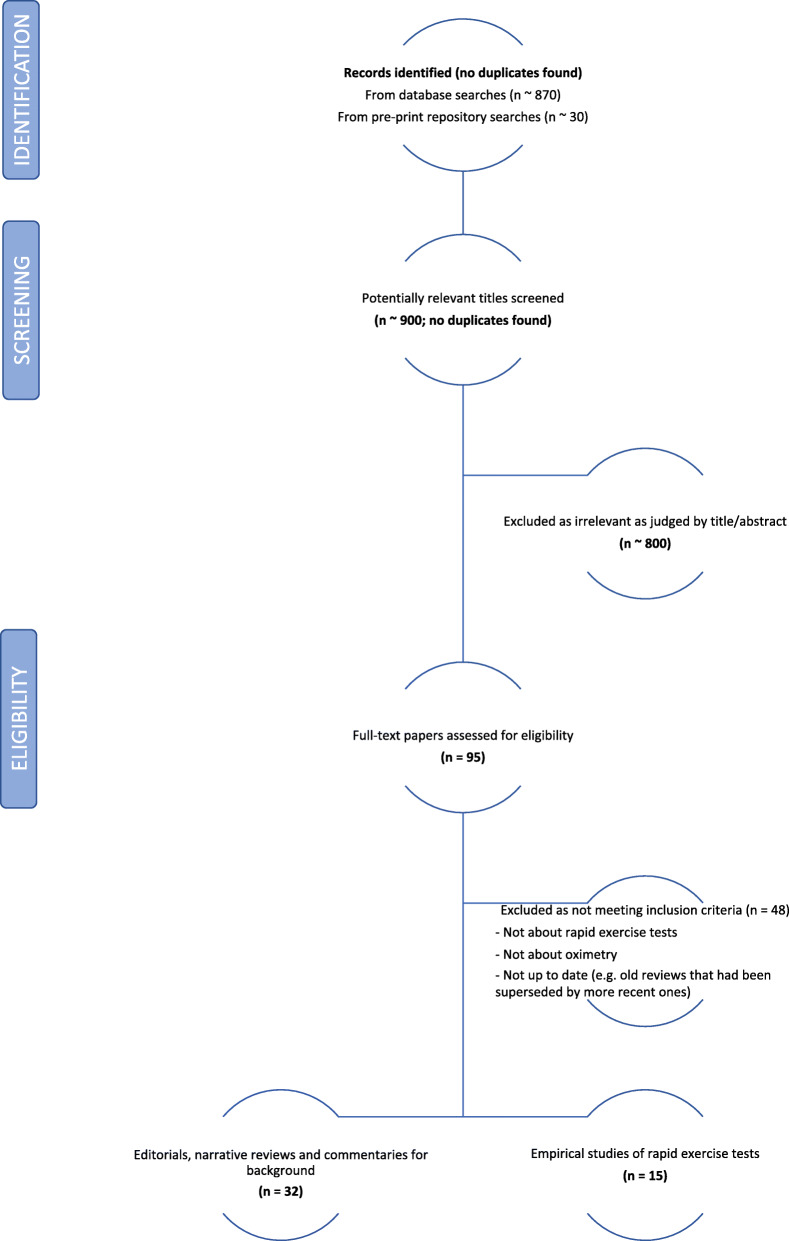
PRISMA flow diagram

The study flowchart is shown in Fig. 1. From approximately 900 potentially relevant articles, we identified 47 relevant papers of which 15 were empirical studies and 32 were narrative reviews, editorials and commentaries. Of the empirical studies, only two (published in 2020) related to patients with Covid-19; neither was a validation study (both considered whether exertional desaturation predicted outcome). Two small studies (published in 1988 and 1994) considered exertional desaturation as a predictive test for pneumocystis pneumonia in people with HIV; we include them because of physiological parallels with Covid-19 discussed below. The other 11 studies were attempts at validation of one or more exercise desaturation tests in chronic lung diseases; they were published between 2007 and 2020 and included between 15 and 107 participants.

The empirical studies are summarised briefly in Table 1 and in more detail in Supplementary Tables S3–S7, which include a detailed risk of bias and applicability assessment. Overall, the methodological quality of most studies as assessed by the QUADAS 2 tool was uncertain. Of the 11 validation studies, none scored ‘high quality’ on all 7 dimensions in the QUADAS2 tool. One (Briand et al 2018 [29]) scored ‘high quality’ in 6 of the 7 dimensions but across the other 8 studies there were many aspects of methodological quality that scored poorly or could not be assessed confidently from the information supplied in the paper. In the sections below, we have placed more emphasis on the studies scoring higher on the QUADAS2 tool and on those which were undertaken on participants with perfusion defects.

The remainder of our results section is divided into four sub-sections. First, we set the context for our review with a narrative summary of the use of exertional desaturation tests in general lung disease. Next, we describe a sparse literature (two studies) in which the results of exertional desaturation tests were correlated with clinical outcomes in Covid-19, followed by an equally sparse literature in which exertional desaturation tests were correlated with the type of pneumonia in HIV-positive patients. Finally, we describe and critique a somewhat larger literature on the validation of selected tests for exertional desaturation in various chronic lung diseases.

### Use of exertional desaturation tests in general lung disease

Tests of exertion in lung disease have mostly addressed the monitoring over time of *chronic* lung disease and have been oriented to measuring exercise capacity. A helpful narrative review by Lee et al. (which drew on an earlier systematic review by European Respiratory Society and American Thoracic Society [30]) lists, for example, a 30-min walk test, a 4-min walk test, a stair climb power test (10 flights), a more moderate stair climb test (un-standardised but based on the patient’s own home stairs), 6-min and 3-min step tests, a 15-step test (step up and down on a 25 cm platform 15 times as fast as possible), Chester step test (an incremental protocol on a 20 cm platform with 2-min phases commencing with 15 steps per minute and increasing by 2 per minute till terminated by dyspnoea or fatigue) and modified Chester step test (starting at 10 steps per minute) [31]. These authors also describe three different sit-to-stand tests: five repetition sit-to-stand (5STS: the patient stands up fully and sits down 5 times as quickly as they can); 1-min sit to stand test (1MSTS: patient stands up fully and sits down as many times as they can in 1 min) and the 30-s and 2-min variants of this [31]. They also review tasks based on activities of daily living such as a semi-standardised ‘grocery shelving task’ [31].

All the exercises described by Lee et al. in the above review were designed mainly for longitudinal monitoring of the severity of chronic lung disease, and several have been shown to correlate with survival [31]. The tests combine an assessment of lung function with that of general physical fitness and muscle strength—a useful composite measure in patients with (for example) chronic obstructive pulmonary disease. They were not originally designed with assessment of acute breathlessness in mind, but as described below, some have subsequently been evaluated for that purpose.

A systematic review considered the validity of the 1MSTST in measuring exercise capacity in patients with chronic lung disease [32]. The main focus of that review was on (a) whether the test correlated with severity of lung disease (broadly, it did), the test-retest reliability of the test (it was high) and whether the test score correlated with the gold standard 6-min walk test (it did). They concluded that ‘The 1-min STS appears to be a practical, reliable, valid, and responsive alternative for measuring exercise capacity, particularly where space and time are limited’ [32]. However, these authors did not look at the 1MSTST in the assessment of exertional desaturation.

The cardiopulmonary exercise test (CPET) has long been used to derive important variables that are known to be good predictors of prognosis in many cardiorespiratory conditions (including chronic obstructive pulmonary disease, interstitial lung disease, pulmonary arterial hypertension, congestive heart failure, cystic fibrosis and chronic thromboembolic pulmonary hypertension) [33]. Several studies have confirmed that peak VO_2_ is the preferred method for risk stratification and for the prognostic evaluation of patients with end-stage lung disease such as COPD and cystic fibrosis [33]. However, field tests (such as the 6MWT and 1MSTST) are more commonly used in clinical practice since they do not require specialist lung function facilities [34].

Fox et al. explored the use of oximetry along with step climbing tests in the detailed assessment of pulmonary capacity, using area under the curve of a continuous oximetry reading [35]. Whilst these authors found that oximetry thus measured correlated with severity of disease and survival, this cannot automatically be applied to the remote assessment of the acutely breathless or hypoxic patient. Similarly, several studies of oximetry in the 6MWT showed strong correlation with disease severity and survival in patients with idiopathic pulmonary fibrosis [36], but these findings may not apply to Covid-19.

### Exertional tests for measuring desaturation in Covid-19

Our search identified no validation studies of exertional tests for hypoxia in patients with Covid-19. We found two studies which sought to correlate the results of an exertional desaturation test with clinical outcomes in Covid-19.

In a small study of 26 COVID-19 patients assessed prior to discharge from hospital, Fuglebjurg et al. used the 6MWT to assess the degree of exertional hypoxia; symptoms of subjective dyspnoea were noted [37]. Thirteen patients developed exercise-induced hypoxia (defined as SpO_2_ < 90%) during the 6MWT, of whom four had pulmonary embolism (a perfusion defect). Covid-19 patients experienced less hypoxia-related dyspnoea during the 6MWT compared with historical idiopathic pulmonary fibrosis controls (none of whom were documented as having pulmonary embolism). The authors concluded that the 6MWT is a potentially useful tool in the diagnosis of asymptomatic exercise-induced hypoxia in hospitalised COVID-19 patients prior to discharge. Whilst interesting, the study does not have direct relevance to the question of exertional desaturation tests in a less select Covid-19 population in the acute phase, nor does it tell us anything about the briefer tests currently in use in community settings.

Goodacre et al. conducted a retrospective observational cohort study (a methodologically weak study design) across 70 emergency departments in the UK during the first wave of the Covid-19 pandemic [38]. Eight hundred seventeen patients out of the 22,000 who were assessed had an exertional test recorded on their record. Of these, 30 had an adverse outcome (defined narrowly as requiring organ support in intensive care) and 9 died. Whilst the positive 1.78 (1.25 to 2.53) and negative 0.67 (0.46 to 0.98) likelihood ratios of a 3% or more desaturation just achieved statistical significance, the authors concluded that exertional desaturation was not a significant predictor of adverse outcome when baseline clinical assessment was taken into account (*p* = 0.37). The study specifically did not assess whether patients with exertional desaturation alone would otherwise have fulfilled criteria for hospital admission. It is possible that if adverse outcome had been more broadly defined (e.g. the need to be admitted to hospital, receive supplemental oxygen or in terms of subsequent healthcare usage), the test may have proved a useful predictor. It is noteworthy that only 3% of the cohort had an exertional test and were not randomly assigned and the reasons for exertional tests being performed/not performed were not analysed. Additional information would have been gained if all patients with a particular baseline oximeter reading had been tested for exertional desaturation and followed up for adverse outcomes. In short, little can be concluded from this retrospective study of a highly selected sample.

### Exertional desaturation as a predictor of acute lung disease

There are some important clinical parallels between pneumocystis pneumonia and the respiratory manifestations of acute Covid-19. Like those with acute Covid-19, patients with pneumocystis pneumonia may be hypoxic (and even cyanotic) on initial presentation, but less severe cases can be normoxaemic initially and become more hypoxic as the disease progresses [39, 40].

In two studies in people with HIV, the value of an exertional desaturation test to discriminate pneumocystis pneumonia from other causes of acute pneumonia was tested. Here, desaturation during the exercise tests was correlated with subsequent results from a bronchoalveolar lavage (and in some cases biopsy) which confirmed or rejected its diagnosis. We describe these studies briefly below.

In a study we rated as high quality, Sauleda and colleagues assessed 45 HIV-positive subjects with pneumonia who were admitted to the emergency department performed pedalling motions in the air for 2 min on the stretcher bed [39]. Oxygen saturations were monitored throughout the test. During exercise, the mean SaO_2_ fell in patients with pneumocystis pneumonia from 88 to 84% (*p* < 0.01), whilst it improved slightly in patient with non-pneumocystis pneumonia from 91 to 93% (*p* < 0.05).

In a similar study (which we rated as lower methodological quality) from 1988 of 39 patients with pneumocystis pneumonia (all HIV-positive young men), exertional desaturation was demonstrated in most of them (including many who had normal saturation at rest) using a 10-min cycling test, whereas patients who presented with other acute lung conditions including bacterial pneumonia, tuberculosis and pulmonary candidiasis were significantly less likely to show exertional desaturation [40].

### Validation of tests for exertional hypoxia in conditions other than Covid-19

We could not find studies comparing different modalities of testing for exertional desaturation in Covid-19. However, we found 11 studies which described attempts to compare and establish non-inferiority of different exercise tests to assess exertional hypoxia in various chronic lung conditions (including chronic obstructive pulmonary disease, interstitial lung disease, advanced lung disease requiring a lung transplant and pulmonary hypertension). Whilst caution must be exercised in applying data from studies in patients with a variety of chronic lung diseases, with differential impacts upon ventilation and diffusion, the data regarding evaluation of tests to detect exertional hypoxia in a variety of conditions and settings is relevant.

In these studies, measured physiological variables from various exercise tests (such as the 1MSTST, 5STST, 2MWT, IST) were compared to those measured with a 6MWT and/or CPET. Variables such as SpO_2_, heart rate and respiratory rate were measured during (and occasionally after) the various exercise tests. Five studies looked at the 1MSTST and showed that it correlated well with the 6MWT and/or the CPET. We describe below the studies in this group which we scored as high quality.

In the study we rated as highest quality, Briand et al. compared the nadir SpO_2_ measured by oximetry on the 6MWT and 1MSTST (performed on the same day) in a clinic population of 107 patients with chronic interstitial lung disease [29]. There was high correlation between the two tests (*r* = 0.9; *p* < 0.0001). The authors also found that the correlation between the tests in terms of desaturation appeared to hold at lower levels of SpO_2_. No adverse events were described in this study. Table 2 shows the distribution of findings in Briand et al’s study.

**Table 2:** Validation study comparing 1MSTST with 6MWT in chronic lung disease

Change in SpO_2_ ≥ 4% in 1MSTST	Change in SpO_2_ ≥ 4% in the 6MWT (gold standard)		
	Yes	No	Total
Yes	42	11	53
No	6	48	54
Total	48	59	107

Data from Briand et al [29]

Using the data in Table 2, and taking the 6MWT as the gold standard, the 1MSTST appears to have a sensitivity of 88% [95% CI impossible to calculate because numbers too small], a specificity of 81% [95% CI 71–91%], a positive predictive value of 79% [95% CI 68–90%] and a negative predictive value of 89% [95% CI impossible to calculate because numbers too small] [29].

Gephine et al. (2020) compared the 1MSTST with the CPET in 14 people with severe COPD and 12 healthy participants [41]. In the COPD group, the fall in SpO_2_ from pre-exercise to peak exercise was similar in the COPD groups with both the 1MSTST (mean − 5% SD 4%) and the CPET (mean − 6%, SD 6%); differences were not statistically significant. In the healthy control group, there was very little fall in SpO_2_ with either the 1MSTST (− 1%, SD 2%) or the CPET (− 1%, SD 1%).

During the 1STST, a ≥ 4% SpO_2_ fall was seen in seven people with COPD, amongst which nadir SpO_2_ values were reached during the recovery period in five patients. For these patients, the lowest value of saturation was reached 33 ± 12 s after the end of the exercise. In comparison, 10 people with COPD exhibited an SpO_2_ fall of similar magnitude during the CPET. In five of them, the SpO_2_ values occurred during the recovery period, 51 ± 16 s after the end of exercise.

The authors concluded that (i) the 1STST elicited a similar peak physiological response to the CPET; (ii) people with COPD showed a nadir SpO_2_ during the recovery period of the 1STST, therefore highlighting the relevance of monitoring this crucial phase of exercise. This study did not, however, report a formal validation exercise of the kind shown in Table 2 (perhaps because numbers were small).

Gloecki et al. found fairly high correlation (*r* = 0.81) between desaturation levels on the 6MWT and those on a shorter 2MWT in a small sample of 26 patients with COPD [42]. Oxygen saturation fell from a mean of 93.8% (95% confidence interval 92.8–94.7) to 83.2% (80.8–85.5) on the 2MWT compared with 93.3% (92.4–94.3) to 82.0% (79.8–84.3) on the 6MWT. Differences in nadir and percentage drop were not statistically significant between the two tests. The authors concluded that ‘the decline in oxygen saturation [is] very similar during the 2MWT and the 6MWT [and] that the short duration of a 2MWT is sufficient to induce a similar oxygen desaturation under room air conditions in patients with severe COPD as the 6MWT’ (page 260) [42].

Rusanov et al. [43] compared the 15SCT against the 6MWT test in 51 patients with idiopathic pulmonary fibrosis (IPF), along with a CPET. SpO_2_ fell from 95% (SD 3) to 86% (SD 7) in the 15SCT and from 94% (SD 3) to 86% (SD 8) in the 6MWT. The nadir of hypoxaemia was very similar on the CPET (88%, SD 6) and showed high correlation with the 15SCT (*r* = 0.85; *p* < 0.0001). The fall in SpO_2_ and nadir SpO_2_ was also highly correlated between 15SCT and 6MWT. The authors concluded that the desaturation measured by the 15SCT test is comparable to the desaturation measured by the CPET and the 6MWD test, making the 15SCT a reliable tool for monitoring disease progression in IPF and for evaluating the need for oxygen supplementation. Another paper by the same authors reports duplicate findings [44]. Again, however, this study only measured *correlation*, without *validation* against the 6MWT and the CPET.

Another study done in patients with a perfusion defect was by Vieira et al., who looked at the usefulness of the IST in 20 patients with pulmonary hypertension [45]. They found a high correlation between desaturation levels on the IST and CPET in patients walking on a treadmill: in the CPET, SpO_2_ fell from 96% (SD3) to 92% (SD 6); in the IST, it fell from 96% (SD 3) to 89% (SD 8)—a difference that was not statistically significant but which may have been clinically significant. The authors concluded that the IST if a useful tool in the evaluation of patients with pre-capillary pulmonary hypertension.

In a further small study of 15 participants with pulmonary fibrosis, Labrecque et al. compared the 1MSTST (done twice to assess reproducibility), 6MWT and CPET [46]. The main aim of the study was to validate the 1MSTST not as a test of exertional desaturation but as a test of exertion which consistently produces a cardiorespiratory stress. 1MSTST, 6MWT and CPET all produced a similar fall in SpO_2_ (10% SD 5, 12% SD 4 and 8% SD 4 respectively). There was no significant difference between the nadir SpO_2_ reached (88% SD4, 85% SD 4 and 87% SD4 respectively), though these differences may have reflected clinically significant differences in desaturation. Perhaps the most important observation from the Labreque study for this review was that the 1MSTST (an intensive burst of exercise over 1 min) was shown to be considerably more strenuous from a cardiorespiratory perspective than the 6MWT and the CPET (which was a longer but less intensive period of exercise lasting 8–9 min). As the authors noted, ‘Coping with such a surge in physiological demand during the 1STS [test] was demanding for people with ILD [interstitial lung disease]’ (page 15).

We placed less weight on the final five studies—as the methodological quality was uncertain, or because methodological quality was considered to be poor on our risk of bias and applicability tool, though the findings from all these additional studies were similar to the above ones. These studies were Gruet et al. (a prospective correlation study from France in 25 patients with cystic fibrosis, which concluded that the 1MSTST may be used as an alternative to the 6MWT and CPET for assessing exertional desaturation) [47]; Morita et al. (a study in 23 patients with COPD which showed good correlation between various exercise desaturation tests) [48]; Kohlbrenner et al. (a retrospective study from Switzerland in 38 lung transplant candidates which concluded that the 1MSTST is a safe alternative to 6MWT in such patients despite lower desaturation nadirs) [49]; Crook et al. (a prospective study in 21 COPD patients which concluded that 1MSTST is a safe and accurate alternative to 6MWT); and Azzi et al. (a retrospective study in 36 lung cancer patients whose main aim was to compare a 3-min chair-to-rise test, 3MCTRT, with the CPET in terms of maximal exercise capacity but which also found high correlation with the level of oxygen desaturation achieved) [50].

The test that is most used in acute practice is the 40-step walk test—the patient is asked to walk 40 steps on the flat and oximetry repeated. We found no research studies on this at all.

## Discussion

### Summary of key findings

This rapid review has produced several key findings relevant to the assessment of exertional desaturation in patients with suspected Covid-19. First, we identified no published studies which had compared the performance of different brief exercise tests in a cohort of patients with (or suspected of having) Covid-19.

Second, in all but one of 11 studies presented as ‘validation studies’ of brief exercise tests for assessing exertional desaturation in other lung diseases, methodological quality was poor or impossible to fully assess. Furthermore, whilst the authors of all 11 studies had *correlated* the level of exertional desaturation on a range of exercise tests with one or both accepted gold standard tests (6MWT and CPET) in various acute or chronic lung conditions, none of these studies had been designed as a formal diagnostic test validation study. Rather, the focus had been on comparing the average SpO_2_ in the same group of patients who underwent both tests and showing no statistical difference between the two measurements. It is reassuring that there was high correlation between the 6MWT or CPET and the shorter 1MSTST, but we would expect the results of these tests to be *correlated* (as, for example, a person’s height will be correlated with their weight). Correlation alone, however, does not *validate* the test [51].

Only one of the 11 studies (Briand et al. [29]) contained sufficient raw data for us to calculate the sensitivity (88%), specificity (81%), and positive and negative predictive value (79% and 89% respectively) of the new test in relation to the gold standard (6MWT), and the authors had not calculated these values themselves. This study suggests that the accuracy of the 1MSTST is far from perfect: 16 patients in every 100 will be misclassified [29]—a finding which underscores the need to interpret them in the context of a full clinical assessment.

The third key finding of this review is that the 1MSTST produced a high cardiorespiratory stress (and indeed, can be exhausting for those who are fully fit), and patients with lung disease continued to show a further drop in SpO_2_ levels even after the test had been completed [46, 52]. In the context of assessing patients remotely who may have acute Covid-19 (i.e. where the clinician is geographically distant from the patient and a full clinical assessment is impossible), the 1MSTST may therefore be risky in unsupervised settings.

Finally, we identified a significant gap in the literature, namely the lack of validation studies (or indeed any relevant research) on the less strenuous 40-step test, which features in local and national guidance for Covid-19 [18] and is in widespread use [19]. This test is likely to be less risky than the 1MSTST but we can currently say nothing about its accuracy. Intuitively, we would expect the specificity to be high, but the sensitivity to be low due to the lower level of physiological exertion required. This is, however, speculative.

### Comparison with previous literature

As the studies listed in Table 1 illustrate, there is a good (though by no means perfect) evidence base in the use of exertional tests for a variety of different pulmonary disease (including airway and interstitial lung diseases). However, the main focus in most such studies is on performance as a surrogate of VO_2_ (for example, noting the number of sit-stands done), rather than oxygen saturation per se. Given Covid-19’s physiological affinity with other perfusion-defect lung conditions such as interstitial lung disease and pneumocystis pneumonia, it follows that the assessment of exertional desaturation would be useful.

As none of the exertional tests have yet been validated to show that assessing exertional desaturation is better than routine clinical assessment at demonstrating increased risk, their use remains pragmatic. The resulting evidence gap in this regard will most likely be addressed in the near future given the continuing incidence of Covid-19.

### Implications for practice

Our review findings, combined with what is known about the pathophysiology of acute Covid-19 [8, 12, 13, 37], suggest a conservative and risk adverse approach to exercise desaturation testing, especially in the home or remote assessment setting. The levels of desaturation observed with brief exercise tests in patients with chronic lung disease [29, 41–43, 45, 46, 48, 49, 52] may be even more marked in those with acute Covid-19. For this reason, we suggest that even a small desaturation on exercise should alert the clinician of the need for further evaluation (such as a face to face consultation) and a drop of 3% should be cause for prompt assessment, regardless of the amount of exercise needed to produce it. There is not yet good evidence that exertional desaturation should prompt a change in treatment (e.g. earlier use of steroids), but this could be the subject of further research.

Current evidence therefore supports the recommendation to undertake a thorough clinical assessment and evaluation including a history, assessment of risk factors, pulse, respiratory rate and oxygen saturations. There is a physiological basis for adding graded assessment into the process of clinical evaluation as a positive test would raise significance.

The ‘40 steps around the room test’ or its alternative ‘40 steps on the spot test’ (in a patient able to stand safely unaided and whose resting saturation is 96% or above) is the lowest level of exertion of any test either in the literature or in clinical practice. An alternative would be the ‘40 steps on the spot test’ with the patient standing in front of a chair in case of needing to sit down. This is appropriate for the home environment (no clinician on hand to guide or resuscitate) given the high-risk group. This test will likely have a low sensitivity but using basic physiological principles, if positive it is likely to be highly specific and warrant urgent assessment.

If the 1MSTS is used, it should be followed by monitoring for at least one minute to observe for desaturation. Indeed, a short test means that it can take time to utilise the free oxygen in the blood stream and therefore witness the impact of desaturation. Such a test should only be done with another person (preferably a clinical staff member) in attendance or nearby. The patient should be kept on continuous oximetry monitoring for at least 1 min following the test; the 1MSTST should never be attempted with a patient home alone.

Our recommendation with regards to oxygen saturation monitoring would therefore be to measure baseline saturations, followed by the 40-step test, followed by the 1 MSTST (if judged safe and there is clinical supervision). If oxygen saturations show a decrease at any of these stages, then the next step should not be attempted.

Oximeters in smartphone apps are unreliable [53], so an approved and tested medical-grade oximeter should be supplied to (or purchased by) the patient. This is happening in some UK localities as part of ‘virtual ward’ arrangements.

Risk to the patient from exercise tests should be considered. Patients should be advised to terminate promptly if they develop any adverse symptoms (severe breathlessness, chest pain, dizziness) [33]. Tests involving climbing a flight of stairs should be avoided, since a staircase is a dangerous place to collapse. A formal 6MWT is not necessary in the home environment but may be useful in following up patients in a clinic or inpatient setting. When doing these more strenuous exertion tests, carefully observe the patient and also make a clinical judgement based on severe fatigue and tachypnoea.

The 40-step test (which is in widespread clinical use but has not been validated) and the 1MSTST are considered the least demanding tests and therefore may be the most appropriate for recommending to patients at home (perhaps modified so the patient is *not* advised to complete ‘as many as you can’ in the 1MSTST). We hypothesise that they are specific but not sensitive (that is, a positive test is serious cause for concern but a negative test should not necessarily reassure), though there is currently no hard data on this.

## Conclusion

A subset of patients with Covid-19 present with marked hypoxia. Earlier identification of these patients to allow closer monitoring and treatment may improve outcome. Exertional desaturation tests are hypothesised to identify these patients before hypoxia at rest occurs. Whilst the evidence base provides some limited support for the equivalence of 1MSTST to the 6MWT or CPET in identifying exertional hypoxia and desaturation, and some comparisons can be drawn to exertional desaturation in PCP, we cannot draw conclusions on the use of these tests within the context of acute Covid-19.

More research is needed on the prognostic value and clinical utility of exertional desaturation tests in all settings (GP, emergency department, ambulance), in the context of Covid-19. Furthermore, an understanding of how best to ask the patient about breathlessness on exertion, and how this correlates with exertion oximetry, could also help in the assessment of hypoxia in Covid-19.

Finally, it is important to remember that a pulse oximeter reading, whether at rest or in an exertional test, does not replace thorough clinical assessment. A normal test does not necessarily mean the patient can be reassured that all is fine. Nevertheless, exertional hypoxia of 3% or more is a significant finding and always warrants further assessment and investigation.

## Supplementary Information

**Additional file 1.**

**Additional file 2.**

**Additional file 3.**

## Data Availability

All sources cited in this review are publicly available.
