# Supplementary material for: Direct and indirect evidence of efficacy and safety of rapid exercise tests for exertional desaturation in Covid-19: a rapid systematic review
Source: Syst Rev. 2021 Mar 16;10:77. doi: 10.1186/s13643-021-01620-w (PMC7961172; doi:10.1186/s13643-021-01620-w)
Supplement: Supplementary file 2 — Additional file 2. [file 13643_2021_1620_MOESM2_ESM.doc]

| **Section/topic** | **#** | **PRISMA-DTA for Abstracts Checklist item** | **Reported on page #** |
| --- | --- | --- | --- |
| **TITLE and PURPOSE** | | |  |
| Title | 1 | Identify the report as a systematic review (+/- meta-analysis) of diagnostic test accuracy (DTA) studies. | See title, page 1 |
| Objectives | 2 | Indicate the research question, including components such as participants, index test, and target conditions. | See research questions in ‘Abstract’, page 2 |
| **METHODS** | | |  |
| Eligibility criteria | 3 | Include study characteristics used as criteria for eligibility. | See methods section in ‘Abstract’, page 2 |
| Information sources | 4 | List the key databases searched and the search dates. | See methods section in ‘Abstract’, page 2 |
| Risk of bias & applicability | 5 | Indicate the methods of assessing risk of bias and applicability. | See methods section in ‘Abstract’, page 2 |
| Synthesis of results | A1 | Indicate the methods for the data synthesis. | See methods section in ‘Abstract’, page 2 |
| **RESULTS** | | |  |
| Included studies | 6 | Indicate the number and type of included studies and the participants and relevant characteristics of the studies (including the reference standard). | See results section in ‘Abstract, page 2 |
| Synthesis of results | 7 | Include the results for the analysis of diagnostic accuracy, preferably indicating the number of studies and participants. Describe test accuracy including variability; if meta-analysis was done, include summary results and confidence intervals. | See results section in ‘Abstract, page 2 |
| **DISCUSSION** | | |  |
| Strengths and limitations | 9 | Provide a brief summary of the strengths and limitations of the evidence | See discussion section in Abstract, page 3 |
| Interpretation | 10 | Provide a general interpretation of the results and the important implications. | See discussion section in Abstract, page 3 |
| **OTHER** | | |  |
| Funding | 11 | Indicate the primary source of funding for the review. | See funding section in Abstract, page 3 |
| Registration | 12 | Provide the registration number and the registry name | N/A |

*Adapted From:*  McInnes MDF, Moher D, Thombs BD, McGrath TA, Bossuyt PM, The PRISMA-DTA Group (2018). Preferred Reporting Items for a Systematic Review and Meta-analysis of Diagnostic Test Accuracy Studies: The PRISMA-DTA Statement. JAMA. 2018 Jan 23;319(4):388-396. doi: 10.1001/jama.2017.19163.

For more information, visit: **www.prisma-statement.org**.

Page 1 of 1
